# Orientation-dependent toxic effect of human papillomavirus type 33 long control region DNA in *Escherichia coli* cells

**DOI:** 10.1007/s11262-020-01754-4

**Published:** 2020-04-03

**Authors:** Eszter Gyöngyösi, Anita Szalmás, József Kónya, György Veress

**Affiliations:** Department of Medical Microbiology, Faculty of Medicine, University of Debrecen, Nagyerdei krt. 98, 4032 Debrecen, Hungary

**Keywords:** Human papillomavirus 33, Long control region, Cloning, Toxicity

## Abstract

The functional analysis of human papillomavirus (HPV) sequence variation requires the molecular cloning of different genomic regions of virus variants. In this study, we report an unexpected difficulty experienced when trying to clone HPV33 long control region (LCR) variants in *Escherichia coli*. Standard cloning strategies proved to be inappropriate to clone HPV33 LCR variants in the forward orientation into a eukaryotic reporter vector (pGL2-Basic). However, by slight modification of culture conditions (incubation at 25 °C instead of 37 °C), constructs containing the HPV33 LCR variants in the forward orientation were obtained. Transformation experiments performed with different HPV33 LCR constructs indicated that there is a sequence element in the 5′ LCR of HPV33 causing temperature-dependent toxic effect in *E. coli*. Sequence analysis revealed the presence of an open reading frame (ORF) in the 5′ part of HPV33 LCR potentially encoding a 116-amino acid polypeptide. Protein structure prediction suggested that this putative protein might have a structural similarity to transmembrane proteins. Even a low-level expression of this protein may cause significant toxicity in the host bacteria. In silico analysis of the LCR of HPV33 and some other HPV types belonging to the species *Alphapapillomavirus 9* (HPV31, 35 and 58) seemed to support the assumption that the ORFs found in the 5′ LCR of these HPVs are protein-coding sequences. Further studies should be performed to prove that these putative proteins are really expressed in the infected host cells and to identify their function.

**Electronic supplementary material:**

The online version of this article (10.1007/s11262-020-01754-4) contains supplementary material, which is available to authorised users.

## Introduction

Genital human papillomaviruses (HPV) belong to the Alpha genus of the *Papillomaviridae* family [1]. Within the *Alphapapillomavirus* genus, the high-risk or oncogenic HPV types (such as HPV16, 18, 31, and 33) are causally associated with the development of premalignant and malignant lesions of the uterine cervix [2]. In certain high-risk HPV types, intra-type sequence variation was shown to be related with variable oncogenic potential [3]. In an effort to explain the differences in the clinical behaviour of intra-type sequence variants, in vitro functional studies have been performed targeting different genomic regions of the HPV variants [4]. The long control region (LCR) of HPV variants has been the subject of several functional studies [5–10].

**Fig. 1:**
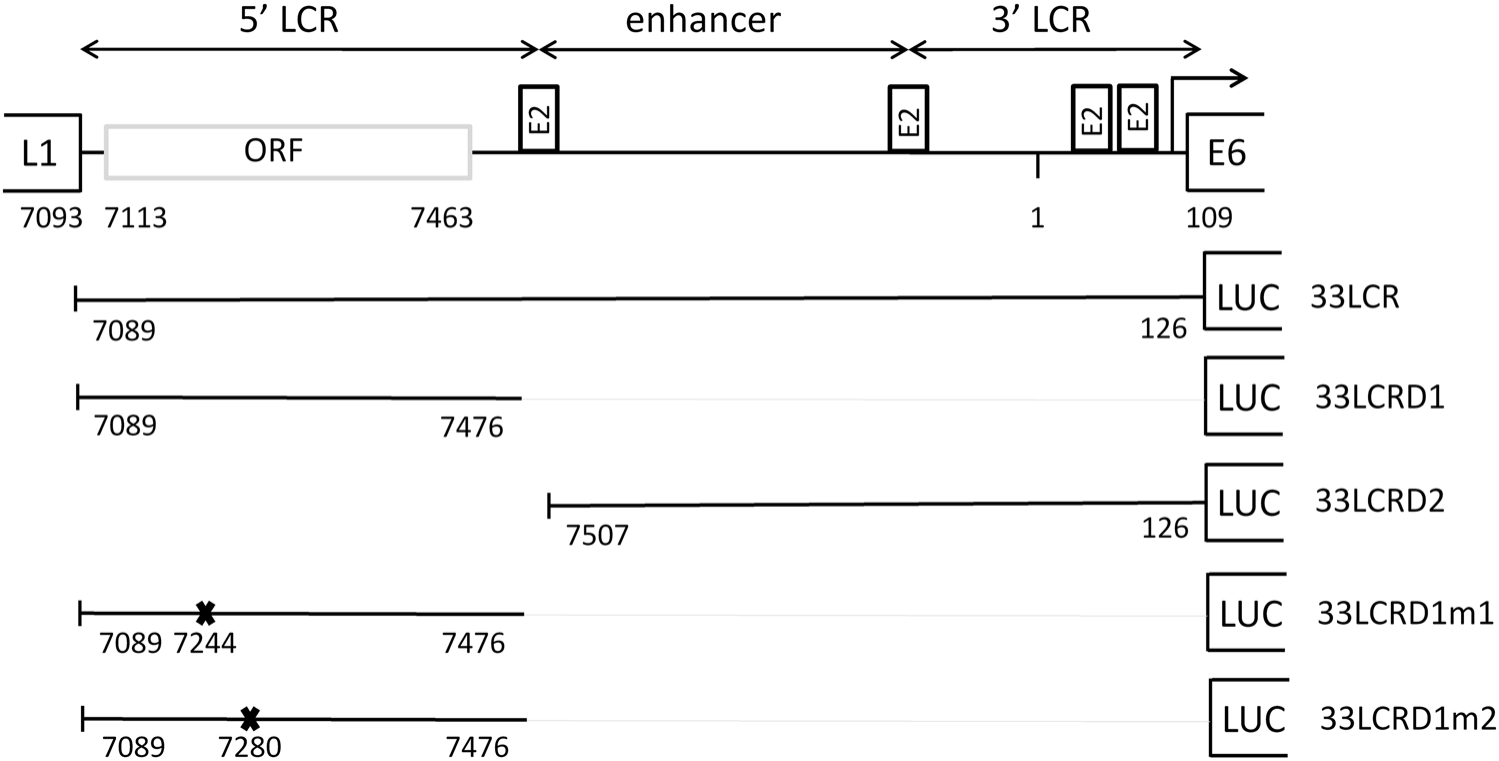
Schematic representation of the different HPV33 LCR constructs used in this study. The locations of the four E2 binding sites and the three functional segments of HPV33 LCR are shown above. A previously unreported ORF found in the 5′ LCR segment is also shown. Numbers indicate nt 1 of the circular genome and the nt positions of the ORFs (shown as rectangles). The reporter constructs shown contain either the full-length HPV33 LCR or different fragments (33LCRD1 and 33LCRD2) or mutants (33LCRD1m1 and 33LCRD1m2) of the LCR each cloned in the forward orientation into the luciferase reporter vector pGL2-Basic

The LCR of HPVs regulates the replication and gene expression of the virus [11]. It contains binding sites for viral and cellular transcription factors. In the *Alphapapillomaviruses*, there are four binding sites for the viral E2 proteins in a conserved arrangement [11]. The LCR is composed of three functional parts (Fig. 1). The 5′ LCR contains a polyadenylation site for the late viral transcripts. The central LCR contains the epithelial-specific enhancer, while the 3′ LCR contains the origin of replication and the promoter for the early viral genes E6 and E7. The LCR of papillomaviruses is not known to encode any viral proteins.

The functional analysis of HPV sequence variation requires the molecular cloning of different genomic regions of virus variants. This is usually a routine task, but in certain HPV types, viral regions or sequence variants may occur that are hard to clone using established methods. Here, we report an unexpected difficulty experienced when trying to clone HPV33 LCR variants in *E. coli*. We show that the HPV33 LCR has an orientation-dependent toxic effect in certain *E. coli* strains. This effect can be localised to the 5′ LCR and might be caused by an open reading frame (ORF) potentially encoding a 116-amino acid polypeptide.

## Materials and methods

### Sample collection, virus isolation, and genotyping

Exfoliated cell samples were collected from the cervix of women with cytological and/or colposcopical abnormalities at the Department of Obstetrics and Gynaecology, University of Debrecen (Debrecen, Hungary). The isolation of viral DNA from the samples was performed with the High Pure Viral Nucleic Acid Kit (Roche, Basel, Switzerland) according to the protocol of the manufacturer. HPV genotyping was performed using the GenoFlow HPV Array Test kit (DiagCor, Kowloon Bay, Hong Kong). Samples found to be positive for HPV33 DNA were selected for detailed sequence analysis of the LCR.

### Polymerase chain reaction (PCR) and cloning

To amplify the whole LCR region of HPV33 variants, the primers 33LCR129 and 33LCR1075 (containing restriction enzyme recognition sites for KpnI and HindIII enzymes, respectively) were used as shown in Supplementary material 1. PCR reactions were performed using the GeneAmp High Fidelity PCR System (Applied Biosystems, Foster City, CA, USA) according to the manufacturer’s recommendations. In 50 µl final reaction volume, 0.1–0.2 µg sample DNA, 5 µl GeneAmp High Fidelity 10X PCR Buffer with MgCl_2_, 500 nM of each primer, 200 nM dNTPs and 2.5 Units GeneAmp High Fidelity Enzyme Mix were included. Amplification was performed on GeneAmp PCR System 2700 (Applied Biosystems). The amplification profile was as follows: 94 °C for 2 min, then 35 cycles at 94 °C for 30 s, 55 °C for 30 s and 72 °C for 60 s. The 72 °C step was increased by 5 s per cycle in the last 25 cycles. After agarose gel electrophoresis, the PCR amplimers were cut from the gels and purified using Qiaquick Gel Extraction Kit (Qiagen, Hilden, Germany). Sequencing of purified PCR products was carried out by UD-GenoMed Medical Genomic Technologies Ltd. (Debrecen, Hungary) using the PCR primers and the Big Dye Terminator v3.1 Cycle Sequencing Kit (Applied Biosystems) and ABI Prism 3100-Avant Genetic Analyzer (Applied Biosystems).

In the first cloning approach, molecular cloning of a representative sequence variant of the HPV33 LCR into the promoterless luciferase reporter vector pGL2-Basic (Promega, Madison, WI, USA) was attempted in an orientation-directed manner. To this end, PCR products, obtained from the LCR of HPV33 and the pGL2-Basic vector were both digested with KpnI and HindIII restriction enzymes and purified with Qiaquick PCR Purification Kit (Qiagen). Ligation was performed with T4 DNA Ligase (New England Biolabs, Ipswich, MA, USA) at room temperature for 2 h. The TransformAid Bacterial Transformation Kit (Thermo Fischer Scientific, Waltham, MA, USA) was used to transform ligation products into XL1, XL1-Blue or DH5α *E. coli* strains according to the protocol of the manufacturer. Culturing of *E. coli* bacteria was performed on Luria Bertani (LB) agar containing 100 µg/ml ampicillin at 37 °C for about 16 h or at 25 °C for about 48 h.

In the second approach (TA cloning), the TOPO TA Cloning Kit with One Shot TOP10 chemically competent *E. coli* cells (Invitrogen, Carlsbad, CA, USA) was used to clone the selected full-length HPV33 LCR variants into a cloning vector (PCR 2.1-TOPO vector). Here, we used similar primers (33LCR129/2 and 33LCR1075/2) and PCR conditions as in the first approach, but the primers contained no restriction enzyme recognition sites. After the PCR reaction, A-addition was performed with Taq DNA Polymerase (Sigma, Saint Louis, MO, USA), then the cloning reaction and transformation (selection with kanamycin at 37 °C or 25 °C) was performed as described above.

To check the orientation of the inserts in the constructs obtained by the TA cloning method, colony PCR was used with primers annealing to the insert (HPV33LCR129/2 or HPV33LCR1075/2) and the cloning plasmid (M13 Forward primer) using REDTaq ReadyMix PCR Reaction Mix (Sigma). The amplification profile used was as follows: 94 °C for 10 min, then 30 cycles at 94 °C for 30 s, 55 °C for 1 min and 72 °C for 1 min 30 s. In the PCR with the primer pair M13 Forward and HPV33LCR129/2, amplimers of around 1,000 bp indicate clones containing the HPV33 LCR in the forward orientation. In the PCR with the primer combination M13 Forward and HPV33LCR1075/2, amplimers of the same length indicate clones containing the insert in the reverse orientation.

To prepare constructs containing deletions in the HPV33 LCR, the same PCR conditions and cloning methods were used as described for directional cloning, except for the primer pairs. To prepare the construct pGL2B-33LCRD1, the primers 33LCR129 and 33LCR516 were used. To prepare the construct pGL2B-33LCRD2, the primer pair 33LCR547–33LCR1075 was used. The template used here was the construct pGL2B-33LCR containing the full-length HPV33 prototype LCR.

Mutagenesis of the pGL2B-33LCRD1 construct was performed using the GeneArt site-directed mutagenesis system (Thermo Fischer Scientific, Waltham, MA, USA) using the primers shown in Supplementary material 1. In the pGL2B-33LCRD1m1 construct, the mutations result in a stop codon at amino acid no. 44 of the putative protein and a change of methionine to leucine at position 45. In the pGL2B-33LCRD1m2 construct, the mutations result in a stop codon at codon 56 and a change of methionine to leucine at codon 57. A schematic representation of different constructs can be seen on Fig. 1. Each plasmid construct was verified by sequencing.

The number of bacterial colonies was counted by the OpenCFU software (https://opencfu.sourceforge.net/) [12].

### Bioinformatics tools

The nucleotide sequences of HPV33 and related HPV types (belonging to the *Alphapapillomavirus 9* species) were obtained from the PaVE (Papillomavirus Episteme) database (https://pave.niaid.nih.gov). In case of HPV31, the sequence was obtained from GenBank (https://www.ncbi.nlm.nih.gov/genbank, accession number HQ537666), because the HPV31 prototype sequence found in PaVE contains sequencing errors in the LCR [6]. The NCBI ORF finder site (https://www.ncbi.nlm.nih.gov/orffinder) was used to search for potential ORFs (open reading frames) in the LCR of the selected HPV types. We searched for ORFs of a minimal length of 150 nt starting with ATG start codon. The LCRs of different HPVs were aligned using MACSE (multiple alignment of coding sequences) software [13]. The MLOGD (Maximum-Likelihood Overlapping Gene Detector) software (https://guinevere.otago.ac.nz/aef/MLOGD/index.html) was used to estimate the probability that the ORFs found in the LCR of certain HPVs might have coding capacity [14, 15]. Protein tertiary structure prediction was performed using the ab initio protein structure prediction tool QUARK (https://zhanglab.ccmb.med.umich.edu/QUARK) [16, 17]. Protein sequence similarity searches were performed using Protein BLAST (https://blast.ncbi.nlm.nih.gov/Blast.cgi). Amino acid sequence alignment of the putative proteins potentially encoded by the LCR ORFs was obtained by the alignment of coding sequences (derived from ORF finder) performed by MACSE. The nucleotide and amino acid sequence alignments provided by MACSE were visualised by SeaView (https://doua.prabi.fr/software/seaview) [18].

## Results

### Cloning of HPV33 LCR may be hampered by the orientation-dependent toxic effect of viral DNA

Most of the clinical samples that were found to be positive for HPV33 with the GenoFlow HPV Array Test kit could be amplified with the HPV33 LCR-specific primers producing amplimers of the expected size. However, an unexpected difficulty was met when trying to clone the PCR products into the promoterless luciferase reporter vector pGL2-Basic using established cloning techniques (including culturing the bacteria at 37 °C). After several trials with different HPV33 LCR variants and different *E. coli* strains (XL1, XL1-Blue, and DH5α), only one clone was found that seemed to contain an insert with the appropriate length, but sequencing of the clone showed that it contained no HPV DNA.

**Fig. 2:**
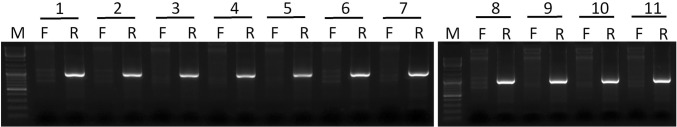
Colony PCR analysis of clones obtained when trying to clone the HPV33 LCR region into the PCR 2.1-TOPO vector using the original protocol (cultivation of bacteria at 37 °C). The different clones analysed by the PCR are indicated by different numbers. F: PCR reaction specific for constructs containing the HPV33 LCR insert in the *forward* orientation. R: PCR reaction specific for constructs containing the HPV33 LCR insert in the *reverse* orientation

Using the TA cloning approach, several clones were obtained that seemed to contain inserts of appropriate length. However, based on the results of orientation-specific PCR tests, all the clones obtained by this approach were found to contain the inserts in the reverse orientation (Fig. 2). This was confirmed by sequencing of selected clones and reproduced several times using different HPV33 LCR variants as PCR templates. This finding was quite unexpected as using TA cloning, one would expect to obtain constructs containing the insert with the forward and the reverse orientation at about the same frequency. If one of the two orientations is not obtained it may refer to the presence of a toxic element in the DNA sequence to be cloned. Therefore, we decided to modify the protocol to reduce the apparent toxicity of the LCR sequence by incubation of bacteria after transformation at 25 °C instead of 37 °C. This simple method was reported to be effective in the construction and propagation of infectious JEV (Japanese encephalitis virus) cDNA clones in *E. coli* [19].

This simple modification of the cloning protocol proved to be very effective also in this case. When bacteria were incubated at 25 °C (for 48 h) after transformation, a higher number of colonies was obtained compared to parallel plates incubated at 37 °C overnight. In addition, about half of the colonies obtained by incubation at 25 °C proved to contain the cloned HPV33 LCR insert in the forward orientation (Supplementary material 2). The constructs obtained by this protocol were found to be stable even after further propagation in broth culture at 25 °C (that was used for plasmid preparation). Sequencing of the constructs confirmed the proper (i.e. forward) orientation of the viral insert in the constructs and showed that no mutations occurred compared to the sequences of the original PCR products.

Next, the directional cloning protocol (cloning of viral insert into pGL2-Basic in the forward orientation) was also modified by incubation of bacteria at 25 °C instead of 37 °C after the transformation step. This modification proved to be very effective also in this case as we were able to clone 6 different HPV33 LCR variants into the reporter vector in the proper (forward) orientation (shown in Supplementary material 3). The constructs were confirmed by sequencing and proved to be stable over time, similarly to the TA cloning protocol. These constructs were also used in luciferase reporter assays performed in human cell lines to study the transcriptional activities of the different HPV33 LCR variants (data not shown here).

### Confirmation and characterisation of the toxic effect of HPV33 LCR on host bacteria

**Fig. 3:**
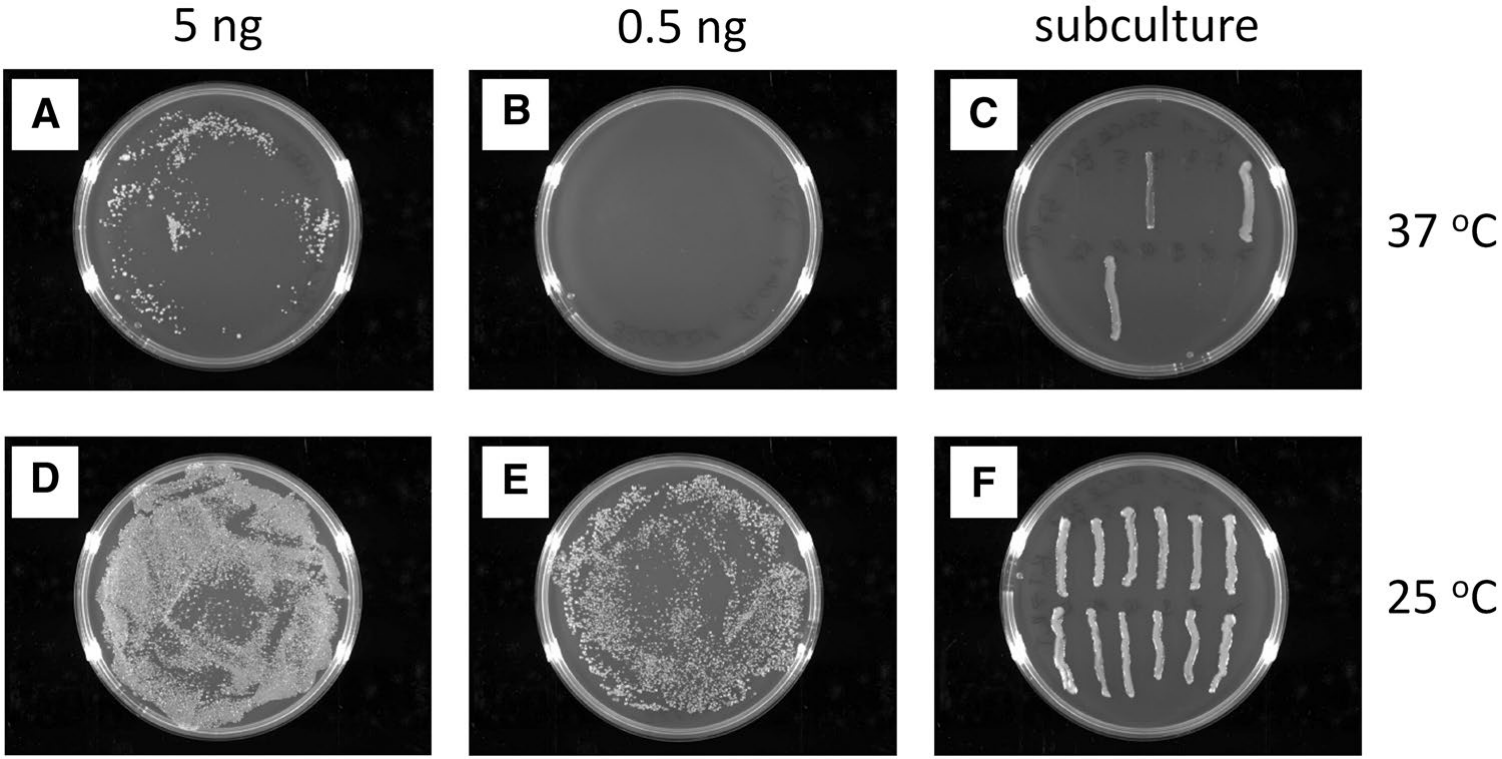
Results of transformation experiments performed with the construct pGL2B-33LCR containing the complete LCR of the HPV33 prototype. XL1 bacteria were transformed by either 5 ng (**a**, **d**) or 0.5 ng (**b**, **e**) of the pGL2B-33LCR construct and incubated at 37 °C overnight (**a**, **b**) or at 25 °C for 48 h (**d**, **e**). C: 12 colonies from the plate shown on A were streaked on a new agar plate and incubated at 37 °C overnight. F: 12 colonies from the plate shown on E were streaked on a new agar plate and incubated at 25 °C for 48 h

Next, transformation experiments were performed to confirm the temperature-dependent toxic effect of the cloned HPV33 LCR DNA on the host bacteria. To this end, XL1 bacteria were transformed by different amounts of a reporter construct containing the whole HPV33 prototype LCR cloned in the forward orientation into the luciferase reporter vector pGL2-Basic (pGL2B-33LCR). After transformation, the plates were incubated at 37 °C overnight or at 25 °C for about 48 h. As shown in Fig. 3, fewer colonies were formed at 37 °C than at 25 °C when plates containing bacteria transformed by the same amount of plasmid DNA were compared. The stability of the clones obtained at different temperatures was tested by sub-culturing on agar plates. Each of the clones formed at 25 °C were found to be stable when sub-cultured at 25 °C, while only a few clones formed at 37 °C could be sub-cultured at 37 °C. These results seem to confirm the toxicity of HPV33 LCR DNA at 37 °C when cloned in the forward orientation into the reporter vector pGL2-Basic.

**Fig. 4:**
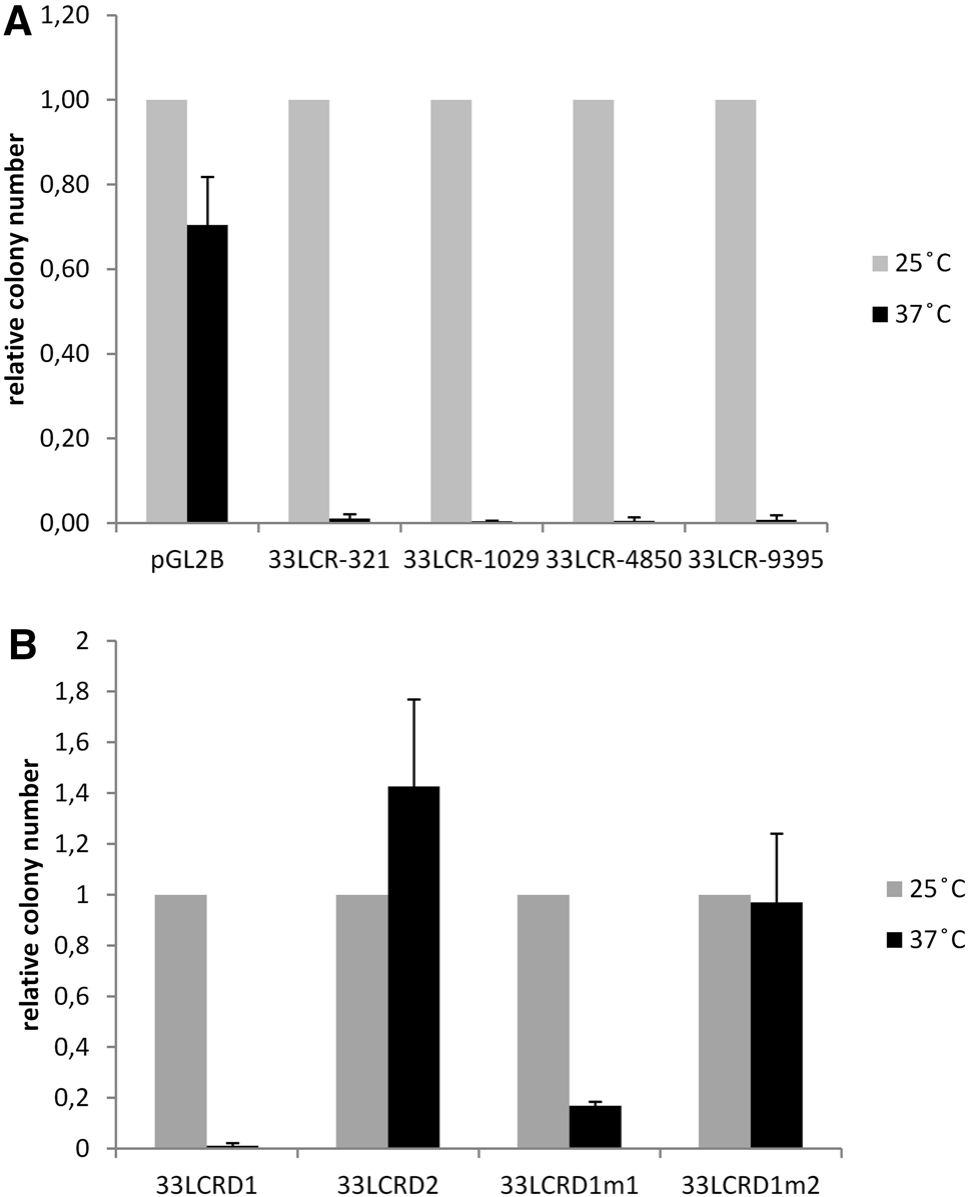
Results of transformation experiments performed with different HPV33 LCR constructs. **a** Results of experiments performed with constructs containing the full-length LCR of different HPV33 variants (shown in Supplementary material 3) cloned into the vector pGL2-Basic. **b** Results of experiments performed with constructs containing different deletions or mutations as shown on Fig. 1. In each experiment, XL1 bacteria were transformed by 500 pg of the different plasmid constructs and incubated at 37 °C overnight or at 25 °C for 48 as indicated. The number of colonies was counted by OpenCFU (https://opencfu.sourceforge.net/). For each construct, the colony number obtained at 25 °C was set to 1 and the colony number obtained at 37 °C is shown relative to this (standardised colony number). The means and standard deviations of 3 independent experiments are shown

To see if the toxicity of the HPV33 LCR prototype sequence is also retained in the natural sequence variants, reporter constructs containing 4 different HPV 33 LCR variants (shown in Supplementary Material 3) were tested in transformation experiments along with the empty reporter vector (pGL2-Basic). As shown in Fig. 4a, each of the constructs containing the different HPV33 LCR variants proved to be highly toxic at 37 °C.

In order to localise the region of HPV33 LCR that is responsible for the toxic effect on the bacterial host, transformation experiments were performed with deletion constructs containing different fragments of the HPV 33 prototype LCR (33LCRD1 and 33LCRD2) (Fig. 1). The results shown on Fig. 4b indicate that the toxic effect of HPV33 LCR can be localised to the region between nt 7089 and 7476 (represented by the 33LCRD1 construct). It is of note that the analysis of the HPV33 reference sequence (obtained from PaVE) performed by ORF finder revealed the presence of a putative ORF in the 5′ part of HPV33 LCR (nt 7113–7463) potentially encoding a 116-amino acid protein (Fig. 1).

Next, two mutants were prepared from the pGL2B-33LCRD1 construct in which the ORF present in the 5′ LCR segment is disrupted by stop codons (Fig. 1). The 33LCRD1m1 construct showed reduced toxicity in transformation experiments compared to the parent construct (33LCRD1), while in the 33LCRD1m2 construct, the mutations disrupting the ORF resulted in a complete loss of toxicity of the LCR fragment (Fig. 4b). Taken together, the results of transformation experiments strongly suggest that the toxic effect of HPV33 LCR DNA on the host bacteria is caused by expression of a protein from the ORF found in the 5′ LCR.

### In silico analysis of the coding capacity of 5′ LCR ORFs found in certain HPV types belonging to the species *Alphapapillomavirus 9*

The ORF finder of NCBI revealed the presence of ORFs of more than 150 nt length (with ATG start codons) in the 5′ LCR of HPV33 and some other HPV types belonging to the species Alpha-9 (HPV31, 35 and 58) (Table 1.). No similar ORFs were found in other HPV types belonging to the species Alpha-9 (HPV16, 52 and 67).

**Table 1:** Positions and characteristics of ORFs found in the 5′ LCR of certain Alpha-9 HPVs

HPV type	Start of LCR	Position of ORF	ORF length	Protein length	MLOGD value^a^
HPV31	7073	7101–7394	294 bp	97 aa	58.07
HPV33	7094	7113–7463	351 bp	116 aa	67.97
HPV35	7110	7143–7316	174 bp	57 aa	38.31
HPV58	7140	7171–7353	183 bp	60 aa	45.95

^a^Total log likelihood ratio summed over phylogenetic tree. Positive values suggest that the tested ORFs are coding

Next, the complete LCRs of these Alpha-9 HPVs (with ORFs in the 5′ LCR) were obtained from PaVE (or GenBank in the case of HPV31) and aligned using the MACSE program [13]. The MACSE alignment tool was chosen because it aligns DNA sequences while considering their coding potential and provides codon-based alignments. The LCR alignment provided by MACSE was analysed by the MLOGD program to estimate the probability that these ORFs are protein-coding sequences (CDSs) [15]. The results of MLOGD analysis indicate that the ORF found in the 5′ LCR of HPV33 is probably a protein-coding sequence (Supplementary Material 4). In addition, the ORFs found in the 5′ LCRs of HPV31, 35 and 58 were also found to be CDSs with high probability as indicated by the high MLOGD values shown in Table 1.

**Fig. 5:**
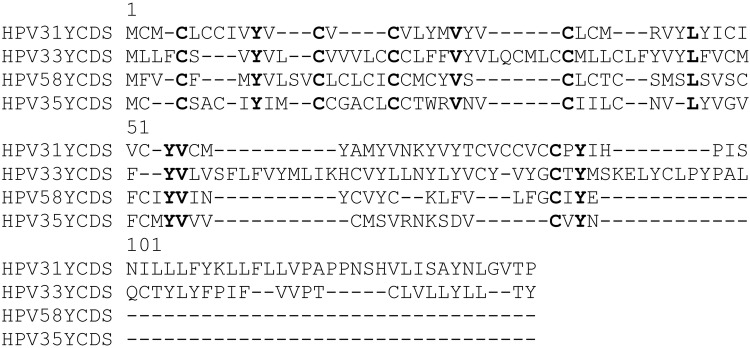
Amino acid sequence alignment of putative proteins potentially encoded by ORFs found in the 5′ LCR of certain Alpha-9 HPVs. The putative coding sequences of ORFs shown in Table 1 were obtained from ORF finder and aligned by MACSE. The protein alignment produced by MACSE was visualised by the SeaView software. Conserved amino acids are shown in bold

Next, the coding sequences of the putative proteins potentially encoded by these 5′ LCR ORFs were obtained using ORF finder, aligned by MACSE and visualised by the SeaView software. As shown on Fig. 5, there is low similarity in the sequences of the putative proteins encoded by the 5′ LCR ORFs of different Alpha-9 papillomaviruses. However, they have a very similar amino acid composition, with overrepresentation of cysteine and hydrophobic amino acids (especially valine and leucine) (Supplementary Material 5). Moreover, there are 5 cysteine residues at conserved positions in all of these putative proteins (in addition to 3 tyrosine, 2 valine and 1 leucine). Sequence similarity search of the putative proteins performed using Protein BLAST revealed no significant similarity to any known viral protein.

Protein structure prediction of the putative proteins encoded by these 5′ LCR ORFs (performed with the help of the QUARK Online tool) showed that they have a similar structure (Supplementary material 6). The putative proteins have 3–4 alpha helices (with loops connecting the helices) and they may have a membrane localisation (because of their highly hydrophobic nature). The putative protein potentially encoded by the HPV35 5′ LCR ORF seems to have a somewhat different structure with 2 short alpha helices and 2 short beta sheets.

## Discussion

The experimental data presented in this article indicate that the HPV33 LCR DNA sequence has an orientation-dependent toxic effect on the host bacteria that can be reduced by lowering the culture temperature to 25 °C. This finding was quite unexpected as the LCR of genital HPVs is not known to code for any protein. In addition, cloning (and functional analysis) of the LCR of HPV33 or of closely related HPV types (HPV16 and 31) has been reported by several research groups, including the group of the authors [5–7, 20–22, 10]. The toxicity of an HPV LCR sequence on the host bacteria may depend on several factors like the presence or absence of an ORF in the LCR, the bacterial strain used for cloning, the cloning vector, and the segment of the LCR cloned (complete LCR or partial sequences). This might explain why this phenomenon has not been reported so far.

The temperature-dependent toxicity of HPV33 LCR in XL1 bacteria was confirmed by transformation experiments (Fig. 3). Not only the prototype HPV33 LCR sequence but also the natural sequence variants tested were found to have significant toxicity on the host bacteria at 37 °C but not at 25 °C (Fig. 4a). The toxic effect of the LCR could be localised to the 5′ LCR segment (Figs. 1, 4b). As this part of the LCR was found to contain an ORF in HPV33 (Fig. 1 and Table 1), it was tempting to speculate that expression of a protein from this ORF may be responsible for the observed toxic effect on the host bacteria. Further reduction of the expression of this putative protein (by culturing at 25 °C) seems to attenuate the toxic activity of the protein.

Transformation experiments performed with mutant constructs indicated that disruption of the 5′ LCR ORF resulted in the reduction or the complete elimination of the toxic effect of the LCR on the host bacteria. Although the ORF encoding the 116-amino acid protein is disrupted in these constructs, some truncated ORFs are preserved potentially providing the expression of smaller proteins. This may be responsible for the remaining toxicity of the 33LCRD1mut1 construct on the host bacteria (Fig. 4b).

The putative product of the ORF found in HPV33 LCR may be a transmembrane protein (see later). The cloning of transmembrane proteins is known to be a challenge because of their toxicity [23, 24]. Therefore, even a low-level expression of this putative protein can be assumed to cause significant toxicity in the host bacteria. However, it is also possible that some other mechanism is responsible for the observed toxic effect of HPV33 LCR, such as the transcription of toxic non-coding RNA sequences. Therefore, further experiments should be performed to identify the exact mechanism that is responsible for the toxic effect of HPV33 LCR DNA on *E. coli* cells.

In silico analysis of the LCR of HPV33 and other HPV types belonging to the species Alpha-9 (HPV31, 35 and 58) seems to support the assumption that the ORFs found in the LCR of these HPVs are protein-coding sequences and may have some functional role in virus infected cells. These ORFs are present at similar locations in all of these four HPV types, between the L1 stop codon and the first E2 binding site (in the 5′ LCR segment). The ORF found in the 5′ LCR of the HPV33 prototype was also conserved in each HPV33 LCR sequence variant analysed in this study (Supplementary material 3). The putative proteins that are supposed to be expressed from these ORFs have a similar amino acid composition (with high percentage of cysteine and hydrophobic amino acids). Although there is relatively low sequence similarity between the putative proteins of different HPV types, they could be aligned and the alignment revealed the presence of several conserved cysteine residues (Fig. 5). The conserved cysteines may be able to stabilise the structure of the proteins (by disulphide bonds) allowing relatively high sequence variation at other positions. This is a well-known characteristic of some cysteine-rich proteins such as defensins [25].

To investigate further the possibility that the ORFs found in the 5′ LCR of these Alpha-9 HPV types are protein-coding sequences, MLOGD analyses were performed using LCR alignment of these HPVs. The MLOGD software compares the probability of coding and non-coding (or single-coding and double-coding) models of a DNA sequence based on the nucleotide and amino acid changes found between the aligned sequences [15]. The results of MLOGD analysis seem to support the assumption that the ORFs found in the 5′ LCR of these Alpha-9 HPV types are protein-coding sequences. Protein structure prediction suggested that these putative proteins have a predominantly alpha-helical structure and probably have a membrane localisation (suggested by the highly hydrophobic nature).

In conclusion, the results presented here indicate that the 5′ LCR segment of HPV33 has an orientation-dependent toxic effect on XL1 bacteria. The toxic effect is probably caused by the expression of a hydrophobic, cysteine-rich membrane protein from an ORF located in the 5′ LCR. In silico analysis of this ORF and similar ORFs found in related HPV types (HPV31, 35 and 58) suggests that these ORFs may be protein-coding sequences. Further studies should be performed to prove that these putative proteins are really expressed in the infected host cells and to identify their function.

## Electronic supplementary material

Below is the link to the electronic supplementary material.

**Supplementary material 1. **PCR and mutagenesis primers used in this study. (PDF 68 kb)

**Supplementary material 2. **Colony PCR analysis of clones obtained when trying to clone the HPV33 LCR region into the PCR 2.1-TOPO vector using the *modified protocol* (cultivation of bacteria at 25 ºC). The different clones analysed by the PCR are indicated by different numbers. F: PCR reaction specific for constructs containing the HPV33 LCR insert in the *forward* orientation.

**Supplementary material 3. **Nucleotide changes found in the different HPV33 LCR variants compared to the reference sequence. (PDF 62 kb)

**Supplementary material 4. **Results of the MLOGD analysis performed with Alpha-9 papillomaviruses carrying 5’ LCR ORFs. The complete LCRs of HPV31, 33, 35 and 58 were obtained from PaVE (or from GenBank in the case of HPV31) and aligned by the MACSE program. The LCR alignment was analysed by the MLOGD operating mode “test input query CDSs” using the HPV33 LCR sequence as the reference. In the analysis shown, the null model was that HPV33LCR contains no CDSs, while the alternate model was that it contains a CDS in the 5’ LCR (nt 7113 - 7463). The log likelihood ratios shown are estimates of the relative probabilities of the alternate model over that of the null model through the analysed region (indicated by the red horizontal bar). (PDF 72 kb)

**Supplementary material 5. **Amino acid composition of putative proteins encoded by the 5’ LCR ORFs of certain HPVs. (PDF 58 kb)

**Supplementary material 6. **Results of the protein structure prediction analysis of the putative proteins potentially encoded by the ORF found in the 5’ part of the certain Alpha-9 HPVs. The analyses were performed using the ab initio protein structure prediction tool QUARK (https://zhanglab.ccmb.med.umich.edu/QUARK/). The amino acid sequences of the putative proteins are shown in Fig. 5. (PDF 356 kb)
