# Supplementary material for: Orientation-dependent toxic effect of human papillomavirus type 33 long control region DNA in Escherichia coli cells
Source: Virus Genes. 2020 Apr 3;56(3):298–305. doi: 10.1007/s11262-020-01754-4 (PMC7220894; doi:10.1007/s11262-020-01754-4)
Supplement: Supplementary file 1 — Supplementary material 1. PCR and mutagenesis primers used in this study. (PDF 68 kb) [file 11262_2020_1754_MOESM1_ESM.pdf]

Supplementary material 1. Primers used in this study.

Primer name	Sequence (5' - 3')[a]	position[b]	application	product name
33LCR129	GCGCGGTACCAATAACACTTTGTGTAATTGTG	7089-7110	directional cloning	pGL2B-33LCR
33LCR1075	GCGCAAGCTTCTCAGTGTCTTGAAACATAGTC	105-126		
33LCR129/2	AATAACACTTTGTGTAATTGTG	7089-7110	TA cloning	pCR2-33LCR
33LCR1075/2	CTCAGTGTCTTGAAACATAGTC	105-126		
33LCR129	GCGCGGTACCAATAACACTTTGTGTAATTGTG	7089-7110	directional cloning	pGL2B-33LCRD1
33LCR516	GCGCAAGCTTTACAATATGGACACTAGTATGT	7455-7476		
33LCR547	GCGCGGTACCTTTCGGTTACTTGGCATACATA	7507-7528	directional cloning	pGL2B-33LCRD2
33LCR1075	GCGCAAGCTTCTCAGTGTCTTGAAACATAGTC	105-126		
33LCR129/2	AATAACACTTTGTGTAATTGTG	7089-7110	colony PCR (forward)	n. a.
M13forward[c]	GTAAAACGACGGCCAG	n. a.		
33LCR1075/2	CTCAGTGTCTTGAAACATAGTC	105-126	colony PCR (reverse)	n. a.
M13forward	GTAAAACGACGGCCAG	n. a.		
33CDS-132FW	GTACTTGTTTGTGTG**AT**TGTTCTATGTACTTG	7229-7260	mutagenesis	pGL2B-33LCRD1m1
33CDS-132RV	CAAGTACATAGAACA**AT**CACACAAACAAGTAC	7229-7260		
33CDS-168FW	TTTCCTGTTTGTGTA**GT**TGTTAATAAAACATT	7265-7296	mutagenesis	pGL2B-33LCRD1m2
33CDS-168RV	AATGTTTTATTAACA**AC**TACACAAACAGGAAA	7265-7296		

[a] Recognition sequences for the restriction enzymes *KpnI* or *HindIII* are underlined. In the sequences of mutagenesis primers, mutated bases are underlined and shown in bold.

[b] The positions of HPV33 specific primers are shown according to the HPV33 reference sequence (GenBank acession no. M12732).

[c] The name of the primer is misleading: this is a reverse primer in this vector.
