# Supplementary figures and images for: Orientation-dependent toxic effect of human papillomavirus type 33 long control region DNA in Escherichia coli cells
Source: Virus Genes. 2020 Apr 3;56(3):298–305. doi: 10.1007/s11262-020-01754-4 (PMC7220894; doi:10.1007/s11262-020-01754-4)

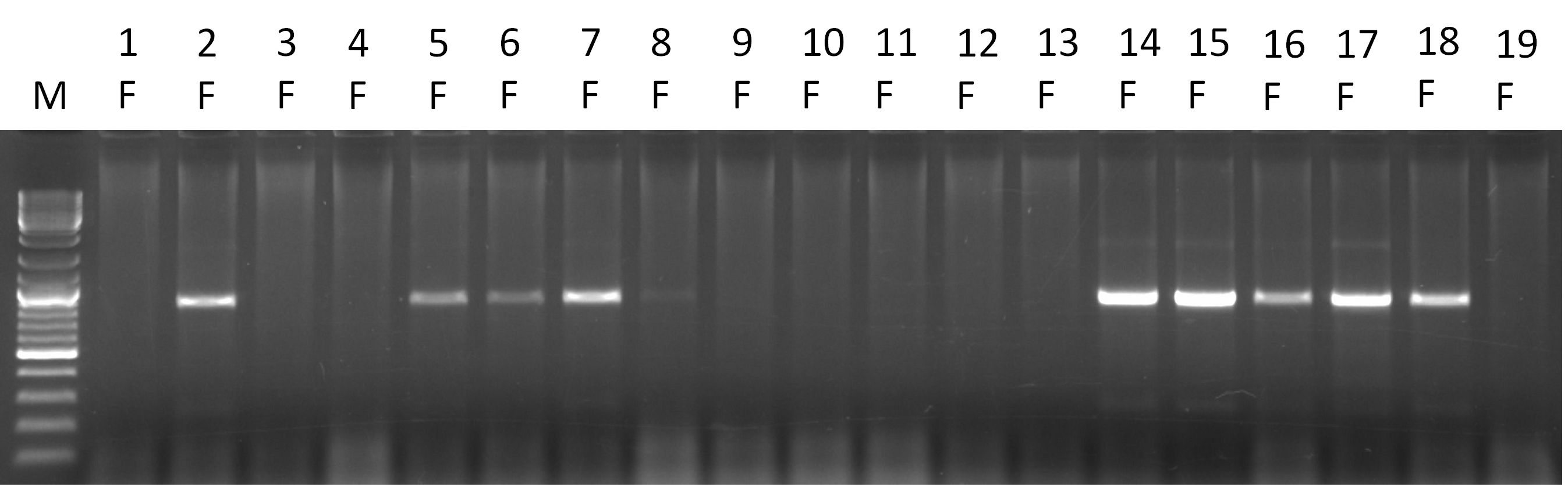

Supplement: Supplementary file 2 — Supplementary material 2. Colony PCR analysis of clones obtained when trying to clone the HPV33 LCR region into the PCR 2.1-TOPO vector using the modified protocol (cultivation of bacteria at 25 ºC). The different clones analysed by the PCR are indicated by different numbers. F: PCR reaction specific for constructs containing the HPV33 LCR insert in the forward orientation. [file 11262_2020_1754_MOESM2_ESM.jpg]
