# Supplementary material for: Orientation-dependent toxic effect of human papillomavirus type 33 long control region DNA in Escherichia coli cells
Source: Virus Genes. 2020 Apr 3;56(3):298–305. doi: 10.1007/s11262-020-01754-4 (PMC7220894; doi:10.1007/s11262-020-01754-4)
Supplement: Supplementary file 3 — Supplementary material 3. Nucleotide changes found in the different HPV33 LCR variants compared to the reference sequence. (PDF 62 kb) [file 11262_2020_1754_MOESM3_ESM.pdf]

Intratypic group	Sample ID	7141	7208	7227	7397	7404	7422	7426	7436	7443	7454	7479	7517-7595	7545	7584	7614	7616	7657	7732	7879	7897	6	18	22	81
	Ref.	T	G	G	C	T	G	C	A	C	G	A	79 bp	G	G	G	C	T	C	A	C	C	G	T	A
**A1**	2015-5009	.	.	.	.	.	.	.	.	.	.	.	.	.	.	.	.	.	.	.	.	.	.	.	C
	8254 *	.	.	.	.	.	.	.	.	.	.	.	.	.	.	.	.	.	.	.	.	.	.	.	C
	2016-321 *	.	.	.	.	.	.	.	.	.	.	.	.	.	.	.	.	.	.	.	.	.	.	.	C
	2016-4979	.	.	.	.	.	.	.	.	.	.	.	.	.	.	.	.	.	.	.	.	.	.	.	C
	2015-9869	.	.	.	.	.	.	.	.	.	.	C	.	A	.	.	.	.	.	.	.	.	.	.	C
	2015-1029 *	.	.	.	.	.	.	.	.	.	.	.	.	.	A	.	.	.	.	.	T	.	.	.	C
	2016-3483	.	.	.	.	.	.	.	.	.	.	.	.	.	A	.	.	.	.	.	T	.	.	.	C
	2016-828	.	.	.	.	.	.	.	.	.	.	.	.	.	A	.	.	.	.	.	T	.	.	.	C
**A2**	2016-4850 *	.	.	A	.	A	T	.	.	.	A	.	DEL	.	.	A	A	.	G	.	.	G	A	.	C
	8995	.	.	A	.	A	T	.	.	.	A	.	DEL	.	.	A	A	.	G	.	.	G	A	.	C
	8053	.	.	A	T	A	T	.	.	.	A	.	DEL	.	.	A	A	.	G	.	.	G	A	.	C
	2016-3082	.	.	A	.	A	T	.	C	.	A	.	DEL	.	.	A	A	.	G	.	.	G	A	C	C
	7851	.	A	A	.	A	T	T	.	.	A	.	DEL	.	.	A	A	.	G	.	.	G	A	.	C
	7929 *	.	.	A	.	A	T	.	.	T	A	.	DEL	.	.	A	A	.	G	G	.	G	A	.	C
	8045	.	.	A	.	A	T	.	.	T	A	.	DEL	.	.	A	A	.	G	G	.	G	A	.	C
	9395 *	.	.	A	.	A	T	.	.	T	A	.	DEL	.	.	A	A	.	G	G	.	G	A	.	C
	8271	.	.	A	.	A	T	.	.	T	A	.	DEL	.	.	A	A	.	G	G	.	G	A	.	C
	8350	.	.	A	.	A	T	.	.	T	A	.	DEL	.	.	A	A	.	G	G	.	G	A	.	C
	7765	.	.	A	.	A	T	.	.	T	A	.	DEL	.	.	A	A	C	G	G	.	G	A	.	C
	2015-4893	G	.	A	.	A	T	.	.	T	A	.	DEL	.	.	A	A	.	G	G	.	G	A	.	C

**AA position**	**10**	**32**	**39**	**95**	**98**	**104**	**105**	**108**	**111**	**114**
Ref. AA	L	M	V	-	Y	V	P	L	L	-
AA change	R	I	M	-	N	L	L	F	F	-

Supplementary material 3. Nucleotide changes found in the different HPV33 LCR isolates at the indicated nucleotide positions compared to the reference sequence (GenBank accession number M12732). Dots indicate the absence of changes relative to the reference sequence at the indicated positions. Amino acid changes that may occur in the putative protein expressed potentially from the ORF found in the HPV33 LCR (nt 7113 – 7463) are shown below. * The indicated isolates were cloned successfully using the modified protocol into the luciferase reporter vector pGL2-Basic.
