# Supplementary material for: Orientation-dependent toxic effect of human papillomavirus type 33 long control region DNA in Escherichia coli cells
Source: Virus Genes. 2020 Apr 3;56(3):298–305. doi: 10.1007/s11262-020-01754-4 (PMC7220894; doi:10.1007/s11262-020-01754-4)
Supplement: Supplementary file 4 — Supplementary material 4. Results of the MLOGD analysis performed with Alpha-9 papillomaviruses carrying 5’ LCR ORFs. The complete LCRs of HPV31, 33, 35 and 58 were obtained from PaVE (or from GenBank in the case of HPV31) and aligned by the MACSE program. The LCR alignment was analysed by the MLOGD operating mode “test input query CDSs” using the HPV33 LCR sequence as the reference. In the analysis shown, the null model was that HPV33LCR contains no CDSs, while the alternate model was that it contains a CDS in the 5’ LCR (nt 7113 - 7463). The log likelihood ratios shown are estimates of the relative probabilities of the alternate model over that of the null model through the analysed region (indicated by the red horizontal bar). (PDF 72 kb) [file 11262_2020_1754_MOESM4_ESM.pdf]

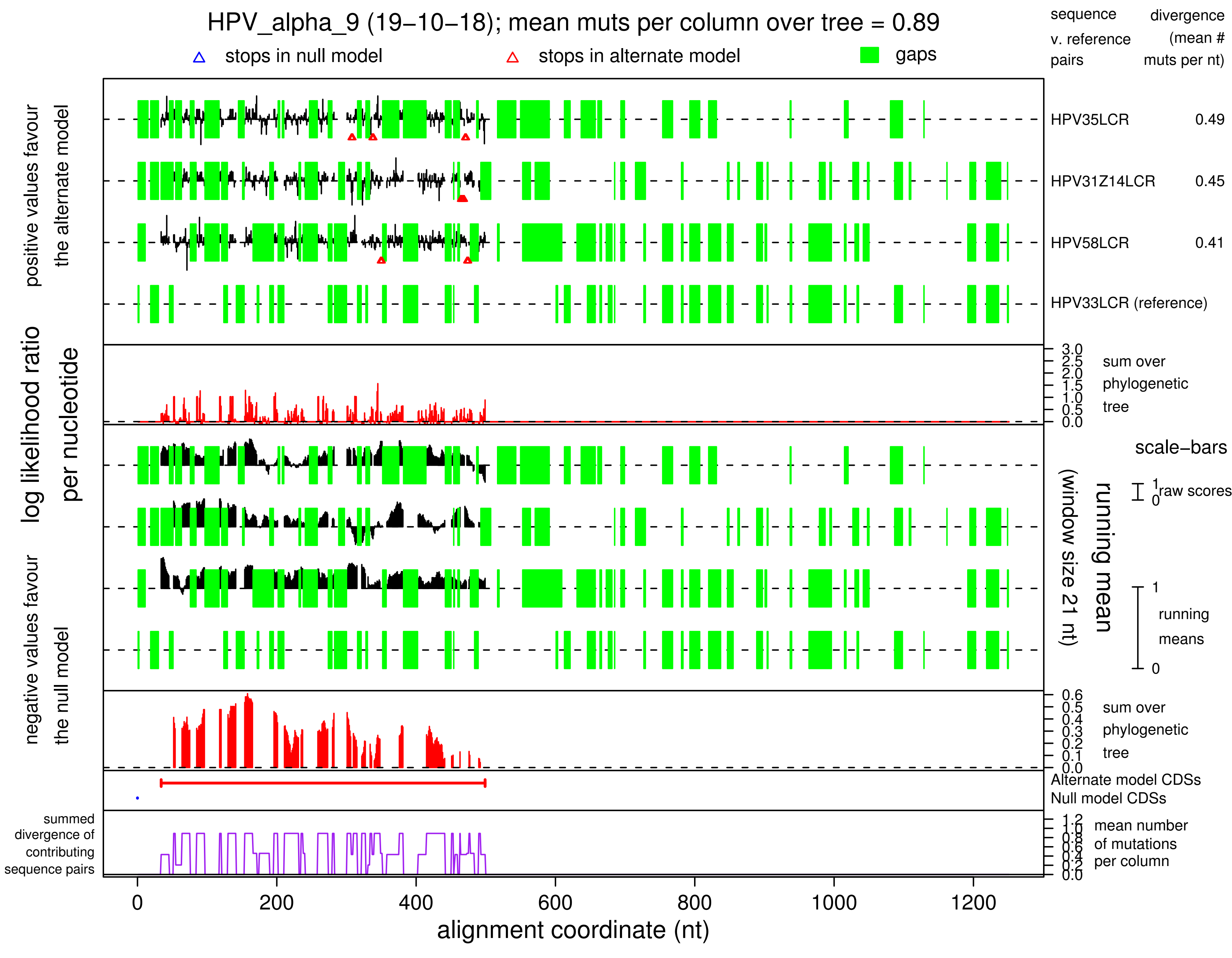

HPV_alpha_9 (19−10−18); mean muts per column over tree = 0.89
stops in null model
stops in alternate model
gaps
sequence v. reference pairs
divergence (mean # muts per nt)
HPV35LCR 0.49
HPV31Z14LCR 0.45
HPV58LCR 0.41
HPV33LCR (reference)
positive values favour the alternate model
log likelihood ratio per nucleotide
negative values favour the null model
sum over phylogenetic tree
scale−bars
raw scores
running means
running mean (window size 21 nt)
Alternate model CDSs
Null model CDSs
summed divergence of contributing sequence pairs
mean number of mutations per column
alignment coordinate (nt)
