# Supplementary material for: Orientation-dependent toxic effect of human papillomavirus type 33 long control region DNA in Escherichia coli cells
Source: Virus Genes. 2020 Apr 3;56(3):298–305. doi: 10.1007/s11262-020-01754-4 (PMC7220894; doi:10.1007/s11262-020-01754-4)
Supplement: Supplementary file 5 — Supplementary material 5. Amino acid composition of putative proteins encoded by the 5’ LCR ORFs of certain HPVs. (PDF 58 kb) [file 11262_2020_1754_MOESM5_ESM.pdf]

	**HPV31Y**	**HPV33Y**	**HPV35Y**	**HPV58Y**
Ala	3,09	0,86	3,51	0,00
Cys	**16,49**	**13,79**	**22,81**	**25,00**
Asp	0,00	0,00	1,75	0,00
Glu	0,00	0,86	0,00	1,67
Phe	2,06	8,62	1,75	8,33
Gly	1,03	0,86	3,51	1,67
His	2,06	0,86	0,00	0,00
Ile	7,22	1,72	7,02	6,67
Lys	2,06	1,72	1,75	1,67
Leu	**13,40**	**22,41**	**5,26**	**11,67**
Met	6,19	5,17	7,02	6,67
Asn	4,12	0,86	7,02	1,67
Pro	6,19	3,45	0,00	0,00
Gln	0,00	1,72	0,00	0,00
Arg	1,03	0,00	3,51	0,00
Ser	3,09	2,59	5,26	10,00
Thr	2,06	3,45	1,75	1,67
Val	**16,49**	**15,52**	**19,30**	**13,33**
Trp	0,00	0,00	1,75	0,00
Tyr	**13,40**	**15,52**	**7,02**	**10,00**
**Total**	**97**	**116**	**57**	**60**

Supplementary material 5. Amino acid composition of putative proteins encoded by the 5' LCR ORFs of certain alpha-9 HPVs. All frequencies are given in percent.
