# Supplementary material for: Orientation-dependent toxic effect of human papillomavirus type 33 long control region DNA in Escherichia coli cells
Source: Virus Genes. 2020 Apr 3;56(3):298–305. doi: 10.1007/s11262-020-01754-4 (PMC7220894; doi:10.1007/s11262-020-01754-4)
Supplement: Supplementary file 6 — Supplementary material 6. Results of the protein structure prediction analysis of the putative proteins potentially encoded by the ORF found in the 5’ part of the certain Alpha-9 HPVs. The analyses were performed using the ab initio protein structure prediction tool QUARK (https://zhanglab.ccmb.med.umich.edu/QUARK/). The amino acid sequences of the putative proteins are shown in Fig. 5. (PDF 356 kb) [file 11262_2020_1754_MOESM6_ESM.pdf]

HPV 31

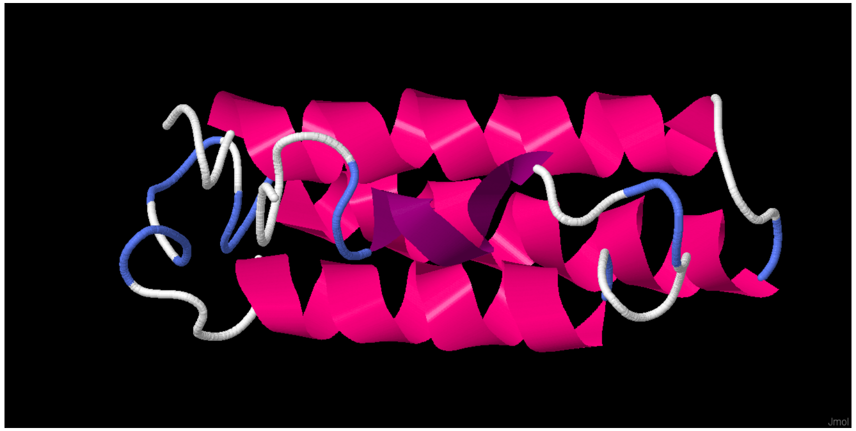

HPV 33

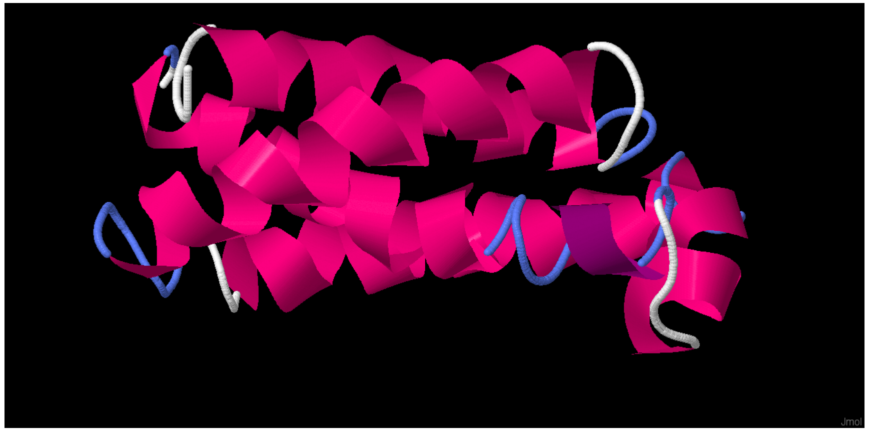

HPV 35

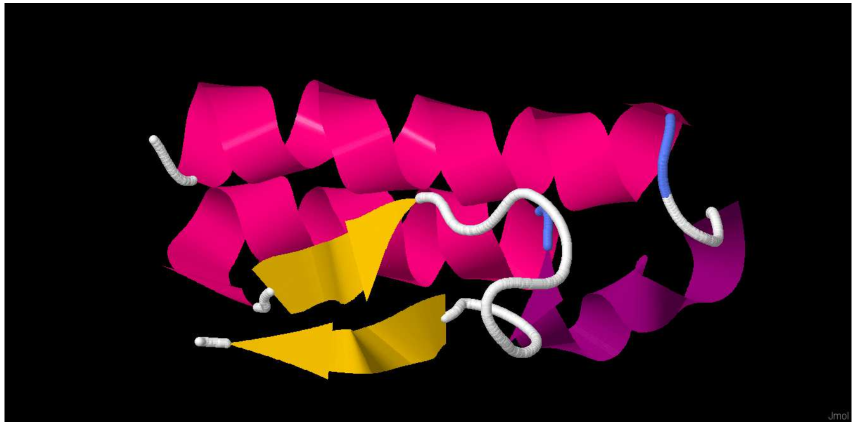

HPV 58

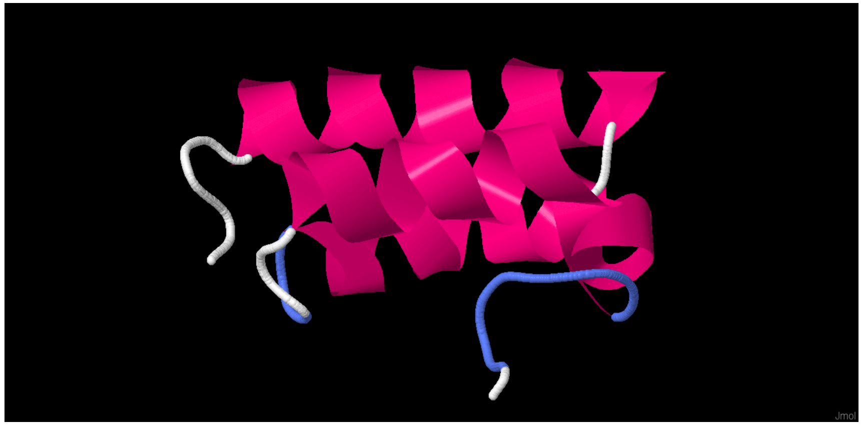

Supplementary material 6. Predicted structures of putative proteins encoded by the 5’ LCR of certain alpha-9 HPVs. Structure predictions were obtained using the QUARK online tool of the Yang Zhang lab (https://zhanglab.ccmb.med.umich.edu/QUARK).
